# Personal protective behaviors in response to COVID-19: a longitudinal application of protection motivation theory

**DOI:** 10.3389/fpsyg.2023.1195607

**Published:** 2023-08-15

**Authors:** Marina Hinssen, Simone Dohle

**Affiliations:** ^1^Gender in Medicine, Charité — Universitätsmedizin Berlin, corporate member of Freie Universität Berlin, Humboldt-Universität zu Berlin, Berlin Institute of Health, Berlin, Germany; ^2^Social Cognition Center Cologne, University of Cologne, Cologne, Germany; ^3^Institute of General Practice and Family Medicine, University Hospital Bonn, University of Bonn, Bonn, Germany

**Keywords:** COVID-19, pandemic, protective behaviors, protection motivation theory, self-efficacy, hand hygiene, physical distancing, mask wearing

## Abstract

**Introduction:**

Disease outbreaks are expected to occur more frequently and spread more rapidly in the age of globalization. Personal protective behaviors strongly affect infection and death rates worldwide. It is therefore of prime importance to better understand which factors predict personal protective behaviors during a pandemic. Protection motivation theory (PMT) proposes that people’s motivation to protect themselves is based on two appraisal processes: threat appraisal and coping appraisal. Building on PMT, this longitudinal study aimed to predict personal protective behaviors in response to COVID-19, including hand hygiene, physical distancing, and mask wearing.

**Method:**

In the first wave of the study (November, 2020), the two appraisal processes as specified in PMT as well as intentions to perform protective behaviors were assessed in a representative sample of German adults (*N* = 328). In the second wave of this study, which was conducted one month later, the frequency of protective behaviors was measured. Structural equation modeling was used to test whether threat and coping appraisal predicted intentions and protective behaviors.

**Results:**

Response rate for the second wave was high (87%). For all three behaviors, self-efficacy predicted intentions and also indirectly behavior (i.e., mediated via intentions). Furthermore, exploratory tests of alternative theoretical models suggested that both self-efficacy and costs have direct effects (i.e., independent from their relationship with intentions) on performed behavior.

**Conclusion:**

To support individuals to engage in protective behaviors during a pandemic, it is important to reduce barriers to action and to foster individuals’ self-efficacy.

## 1. Introduction

Even in the third year after the novel coronavirus disease 2019 (COVID-19) emerged, the subsequent pandemic can hardly be considered defeated. Albeit most vaccines are effective at preventing disease and hospitalization, the likelihood of breakthrough infection remains moderately high and even patients with a mild disease progression can suffer from persisting symptoms months after their acute infection (Robert-Koch-Institute, 2022). These factors make it vital for individuals to follow public health guidelines to protect themselves and contain the spread of the virus (Robert-Koch-Institute, 2022). For a wide range of health behaviors, Rogers’ protection motivation theory (PMT) has proven a valuable framework to infer their determinants (Rogers, 1975, 1983; Milne et al., 2000). This longitudinal study tests whether it can be applied to explain adherence to COVID-19 preventive measures in a nationally representative sample of the German adult population. It aims at identifying factors that might affect peoples’ intention to adopt protective measures as well as their actual behavior. In investigating social cognitive variables amenable to change in interventions, our study may provide guidance to health campaigning efforts during the ongoing pandemic and any future disease outbreak (Rogers, 1975, 1983; Milne et al., 2000).

### 1.1. Protection motivation theory

The major assumption of PMT is that, once individuals receive information about a threat to their health (e.g., the risk of an infection with COVID-19), two cognitive processes are initiated that ultimately determine the probability of showing an adaptive coping response to the threat (Rogers, 1983). One of these processes, *threat appraisal,* comprises the following three components: (a) the assessment of the likelihood of being personally affected (*perceived vulnerability*), (b) the evaluation of magnitude of negative consequences (*perceived severity*), and (c) emotions of fear that are evoked by the threat *(fear arousal)*. *Coping appraisal*, on the other hand, subsumes (a) beliefs about the efficacy of the recommended response to effectively avert negative consequences *(response efficacy)* (b) beliefs about one’s capability to perform the recommended protective behaviors *(self-efficacy)* and (c) the expected personal costs of adopting these *(response costs).* High threat perceptions (i.e., high levels of perceived severity, vulnerability, and fear arousal) and positive evaluations of the recommended coping responses (i.e., high levels of response efficacy and self-efficacy; expectation of low response costs) are proposed to increase an individual’s intention to engage in protective behaviors (*protection motivation*). Through their effect on intention, they are hypothesized to indirectly increase the likelihood that protective measures are actually taken.

Earlier research conducted during past pandemics (e.g., SARS-CoV-1, avian influenza/flu H5N1, swine influenza/flu H1N1) and the current COVID-19 pandemic lends initial support to the proposed bivariate associations of the six appraisal components with intentions and performed protective behaviors. Specifically, cognitive risk perception and the experience of fear or worry due to a specific infectious disease have been shown to be positively related to reported intentions and protective behaviors (Bish and Michie, 2010; Rubin et al., 2010; Dryhurst et al., 2020; Coifman et al., 2021). Furthermore, individuals reported stronger intentions and more thorough adherence when they believed that the different protective measures were effective at reducing their risk of contracting a certain disease (Rubin et al., 2010; Bults et al., 2011; Kaspar, 2020; Kowalski and Black, 2021; Scholz and Freund, 2021; Van Loenhout et al., 2021) and were confident in their own ability to do so in everyday life (Kaspar, 2020; Kowalski and Black, 2021; Scholz and Freund, 2021; Van Loenhout et al., 2021). On the contrary, expecting the measures to be costly, e.g., in terms of time and effort needed for hygiene and distancing practices, was associated with lower intentions and adherence (Al-Rasheed, 2020; Kaspar, 2020). In many of these studies, effects were stronger and more consistent for the coping variables as compared to the threat variables (Farin, 1994; Teasdale et al., 2012; Kaspar, 2020; Kowalski and Black, 2021; Scholz and Freund, 2021; Van Loenhout et al., 2021).

Research in light of pandemic events has oftentimes been carried out as a rapid response to emerging influenzas (Rubin et al., 2010), characterized by profound methodological weaknesses and a lack of a theoretical framework (Leppin and Aro, 2009). For the most part, data has been collected through convenience sampling or by surveying specific subgroups of the population (e.g., inhabitants of a certain region, students, or medical staff) (Al-Rasheed, 2020). This poses problems to the generalizability of results to the general public (Kaspar, 2020; Van Loenhout et al., 2021). An even greater concern is the predominance of cross-sectional surveys casting doubt on causal inferences about the effects of the appraisal processes on subsequently formed intentions or performed behaviors (Brewer et al., 2004; Norman et al., 2015).

### 1.2. The case for model modification

Protection motivation theory provides a comprehensive description of factors that might influence the motivational process of deliberation, choice of goals and corresponding actions (Norman et al., 2015). Intentions are proposed to be the main driver and sole predictor of performed protective behaviors (Norman et al., 2015). Although meta-analytic work shows that there is a strong relationship between intentions and subsequent health behavior (*r* = 0.53), this means that 70% of variance are typically not explained (Sheeran, 2002). An individual’s motivation, therefore, does not seem to sufficiently explain whether or not he or she is taking health protective action (Sheeran, 2002). We propose that, once an intention to adopt preventive measures is set, the two PMT-constructs self-efficacy and perceived costs continue to play a role in determining its successful implementation. As the authors of other prominent social cognitive theories [e.g., the *theory of planned behavior/reasoned action approach* (Ajzen, 1991; Schwarzer, 2008; Fishbein and Ajzen, 2009)] point out, the belief in one’s capability of performing a behavior (i.e., self-efficacy) is likely to remain crucial for behavioral initiation and maintenance after an intention has been set, especially when difficulties arise and setbacks have to be overcome. Relatedly, perceived costs can reflect those difficulties [i.e., inconvenience, discomfort, financial expense, disruption of everyday life (Rogers, 1983)] that might be subjectively experienced in the very moment a protective behavior needs to be carried out. Visceral drives–e.g., aversive experiences such as hunger, fatigue, and negative emotions–play an important role in explaining impulsive risk behaviors that are in conflict with individuals’ long-term health goals (Loewenstein, 1996, 2005; Hofmann et al., 2008; Nordgren et al., 2008). As powerfully demonstrated by Nordgren et al. (2008), those temporary states affect individuals’ health cognitions and behavioral intentions: Hungry dieters and smokers with a momentary cigarette craving follow less ambitious weight loss goals and intentions to quit smoking, respectively, than did their satiated or noncraving counterparts. Furthermore, the latter were more confident in their ability to reach their goals. Their greater optimism reflects the well-documented phenomenon of the *cold-to-hot-empathy gap*: When in a “cold state” or not affectively aroused (e.g., not hungry), people seem to systematically underestimate the intensity of future aversive states and their impact on their own preferences and behavior in the future (Loewenstein, 1996, 2005). Likewise, people who are currently unaffected by the burden of COVID-19 protective behaviors (e.g., the discomfort of wearing a facemask) might underestimate the motivational force of those costs when reporting their intentions to adhere, yet be strongly influenced by them in situations critical for infection protection. Thus, perceived costs might have a strong influence on performed behavior, whereas their influence on intentions might be less pronounced.

Besides their psychological effects, both self-efficacy and perceived costs might reflect the amount of actual control people possess over performing a behavior based on their actual abilities, opportunities, and the existence of actual barriers to action (Ajzen, 1991; Fishbein and Ajzen, 2009). Such non-motivational factors may affect behavioral performance independently from a person’s intentions (Fishbein and Ajzen, 2009). In fact, several PMT-based studies found self-efficacy to be directly predictive of health behavior along with intention (Conner and Norman, 2015; Dowd et al., 2016). The direct effect of costs on behavior can possibly be inferred from *nudging* interventions that proved successful in improving health behavior (Sniehotta et al., 2014; Bucher et al., 2016). In this domain, nudging refers to a strategic change in the choice architecture aimed at making healthy choices easier, e.g., by making the respective option psychologically salient or appealing or reducing the option-related effort (Bucher et al., 2016; Hansen et al., 2016). For example, placing fruit within consumers’ reach has been shown to positively affect its intake (Bucher et al., 2016). Low costs─favorable environmental conditions and the absence of barriers to actions─may therefore contribute to health protective behaviors without affecting intention or self-efficacy (Sniehotta et al., 2014). Based on the aforementioned arguments and empirical findings, a modification of the PMT model seems to be necessary: Besides intention, self-efficacy and perceived costs might also directly predict performed protective behavior (Fishbein and Ajzen, 2009; Conner and Norman, 2015).

### 1.3. The present study

The main aim of this study was to test the utility of Rogers’ PMT for better understanding protective behaviors in the COVID-19 pandemic. Expanding on past work, we present the results of a longitudinal survey in a sample representative for age and gender of the German adult population. The longitudinal survey design and the representative sample (stratified by age and gender of the German adult population) address main weaknesses of existing research and reduce ambiguity in causal interpretation of the findings (Brewer et al., 2004; Norman et al., 2015; Al-Rasheed, 2020; Kaspar, 2020; Van Loenhout et al., 2021).

Existing studies during pandemics investigated one particular prevention measure as outcome variable (e.g., either hand washing or facemask wearing) or combined multiple different behaviors into one scale measuring overall adherence (Bish and Michie, 2010; Hagger et al., 2020). Here, we aim to explain the adoption of the three “DHM-measures”–keeping physical distance (“D”), proper hand hygiene (“H”) and wearing a face mask (“M”)–which were promoted by the German Federal Centre for Health Education since the beginning of the COVID-19-pandemic (Federal Centre for Health Education, 2020). Since these behaviors differ in many aspects (e.g., in terms of discomfort, frequency, novelty, and impact on others) it is to be expected that different cognitions determine their performance (Bish and Michie, 2010). By specifying three SEMs for each of these behaviors, we aim to identify more precisely which of the PMT constructs might be specifically promising target variables to promote each of them individually.

In line with PMT, we hypothesized that higher levels of perceived severity of the disease, of perceived personal vulnerability to an infection, and of experienced fear arousal would have a positive effect on an individuals’ intention to adopt protective measures at the baseline measurement. Second, both perceived response efficacy and self-efficacy beliefs concerning the performance of the behaviors in everyday life were expected to have a positive effect, whereas perceived costs were hypothesized to reduce the respective behavioral intentions. We also hypothesized that these factors would have an indirect effect on actual behavior performed in the following month through their influence on intentions. That is, severity, vulnerability, fear arousal, self-efficacy and response efficacy were expected to increase the actual adoption of protective behavior via increased behavioral intentions. Costs, on the other hand, were assumed to be negatively associated with performed protective behavior *via* its inhibiting effect on intentions. In an additional exploratory investigation, we tested a second, integrated social cognition model designed to illuminate the implementation of adherence intentions more comprehensively. Our proposed model alternative incorporated two additional direct effects of both self-efficacy and costs on behavior.

## 2. Methods

### 2.1. Sample

Data were collected in two consecutive surveys waves in mid November and mid December 2020. The study thus was conducted at the beginning of the second wave of the COVID-19 pandemic in Germany, after an exponential surge of confirmed cases had been observed in October 2020 (Schuppert et al., 2021; Robert-Koch-Institute, 2022). Starting from November, new containment measures of partial lockdown were implemented by the German government that primarily aimed at restricting leisure time activities (i.e., closing of restaurants, cinemas, and theaters) and contacts (i.e., banning gatherings of members of more than two households in public) (Schuppert et al., 2021). On the day the first assessment (Time 1 [T1]; 13.11.─19.11.2020) started, 751,095 cases and 12,200 deaths due to COVID-19 had been reported in Germany (Robert-Koch-Institute, 2022). Since growth of infections remained strong in November and December, lockdown measures were extended by the closure of schools, day care centers and retail shops on December 16, 2020 (Schuppert et al., 2021). On the first day of the second survey wave (Time 2 [T2]; 14.12─20.12.2020), there had been 1,351,510 confirmed cases and 22,475 deaths related to COVID-19 in Germany (Robert-Koch-Institute, 2022).

A nationally representative sample of the German adult population (stratified by age and gender) was recruited by the panel provider Respondi^1^ and invited to complete the survey hosted on Qualtrics.^2^ Out of *N* = 467 individuals who started the survey, *n* = 8 refused to give their consent. *n* = 30 dropped out before completing the questionnaire, and *n* = 97 were excluded due to a failed attention check (“Please mark the response option 1”). In a series of items presented to detect careless responses (Meade and Craig, 2012), *n* = 3 individuals indicated that we should not use their data for analyses. We removed their records as well as the second record of one participant whose ID indicated duplicate submission. When the final sample at T1 (*N* = 328) was contacted again one month later at T2, *n* = 285 individuals (87%) opened the follow-up questionnaire, out of which *N* = 278 gave their consent and fully completed the survey. The mean age of this final sample was 45.83 years (*SD* = 14.57), and 50.7 percent were female. Detailed demographic characteristics of the T1 and T2 samples are displayed in Supplementary Table A1 in the Supplementary material for this article. Note that merging data into a longitudinal data file revealed eight persons with inconsistent or implausibly altered indicator variables across waves (age, gender). We decided to keep their records in the sample as no obvious signs of poor-quality responses (e. g. speeding, response sets) could be detected.

### 2.2. Measures

Item examples and scale statistics can be found in Table 1 (see Supplementary Tables A2–A4, for a translated version of all measures). The Time 1 questionnaire measured threat (severity, vulnerability, fear) and coping (response efficacy, costs) appraisals as well as behavioral intentions. Four weeks later, performed behavior was assessed. Measures were based on items used in previous work on PMT (Milne et al., 2000) and adapted to the adoption of protective behavior during the pandemic. For each of the three DHM-behaviors, separate sets of items were presented to assess coping appraisal (response efficacy, self-efficacy, perceived costs), intentions, and behavioral frequency. Within these sets, each item referred to the adoption of either distancing, hand hygiene, or mask-wearing behavior in specific everyday life situations that have been emphasized to be especially important for prevention in public health campaigns (Federal Centre for Health Education, 2020). For example, looking at perceived response efficacy of wearing a mask, participants would indicate whether they believed that it would reduce their risk of an infection while (a) using public transport, (b) spending time in public places, (c) meeting other people and (d) entering supermarkets or other shops.

**Table 1:** Overview of variables and psychometric data (ranges, number of items, construct reliabilities).

Construct	Behavior	Item example	No. of items	*ρ*
Severity	D	How severe would the consequences of an infection with the coronavirus be for your personal health?	4	0.95
	H		4	0.95
	M		4	0.95
Vulnerability	D	It is very likely that I am getting infected with the coronavirus until the end of the year.	3 (4)	0.94
	H		3 (4)	0.95
	M		3 (4)	0.94
Fear	D	The thought of getting sick from the coronavirus makes me feel anxious.	4	0.98
	H		4	0.98
	M		4	0.98
Response efficacy	D	Keeping a distance of at least 1.5 meters from other people while entering supermarkets or other shops markedly reduces the risk of an infection with the coronavirus.	4 (5)	0.97
	H	Washing one’s hands thoroughly (for at least 20 s with soap) when coming home markedly reduces the risk of an infection with the coronavirus.	4	0.94
	M	Wearing a face mask (hygienically clean and fully covering mouth and nose) when meeting other people from different households markedly reduces the risk of an infection with the coronavirus.	3 (4)	0.97
Self-efficacy	D	I am confident in my ability to, in the next four weeks, always and under all circumstances, keep a distance of at least 1.5 meters from other people when I am in supermarkets or other shops.	4 (5)	0.93
	H	I am confident in my ability to, in the next four weeks, always and under all circumstances, wash my hands thoroughly after coming home, i.e., for at least 20 s with soap.	4	0.84
	M	I am confident in my ability to, always and under all circumstances in the next four weeks, wear a hygienically clean face mask fully covering mouth and nose at longer meetings in small spaces with other people from other households.	3 (4)	0.93
Costs	D	It is uncomfortable/exhausting/can cause difficulties to keep a minimum distance of 1.5 meters to other people.	3	0.9
	H	It is uncomfortable/inconvenient/can cause difficulties to wash one’s hands frequently and thoroughly for at least 20 s with soap.	3	0.88
	M	It is uncomfortable/inconvenient/can cause difficulties to wear a face mask (hygienically clean and fully covering mouth and nose).	3	0.9
Intention	D	I intend to, in the next four weeks, always and under all circumstances, keep a distance of at least 1.5 meters from other people when I am in supermarkets or other shops.	4 (5)	0.83
	H	I intend to, in the next four weeks, always and under all circumstances, wash my hands thoroughly after coming home, i.e., for at least 20 s with soap.	4	0.83
	M	I intend to, in the next four weeks, always and under all circumstances, wear a hygienically clean face mask fully covering mouth and nose at longer meetings in small spaces with other people from other households.	3 (4)	0.9
Behavior	D	During the last four weeks I, under all circumstances, kept a distance of at least 1.5 meters from other people in supermarkets or other shops.	4 (5)	0.73
	H	During the last four weeks I, under all circumstances, washed my hands thoroughly after coming home, i.e., for at least 20 s with soap.	4	0.79
	M	During the last four weeks I, under all circumstances, wore a hygienically clean face mask fully covering mouth and nose at longer meetings in small spaces with other people from other households.	3 (4)	0.73

Presented numbers of items refer to the numbers of indicators used in our final measurement models. Written in brackets are the numbers of items before removal of those items deemed inappropriate (see CFA). Raykov’s rho coefficient (*ρ*) based on *N* = 275 for physical distancing (D) and wearing of a face mask (M), based on *N* = 271 for hand hygiene (H). For the assessment of perceived severity, participants assessed the consequences on a 7-point scale (1 = no negative consequences, 7 = extreme negative consequences). All other constructs were measured on a 7-point scales ranging from 1 = disagree strongly to 7 = agree strongly.

Regarding demographics, we collected participants’ age and gender in both survey waves. At Time 1 only, they were asked to report their subjective health status (1 = *very bad*, 5 = *very good*), whether a medical condition put them at greater risk of severe illness from COVID-19, and whether the latter applied to a significant other. Additional questions tapped into the endorsement of conspiracy theories, trust in politics and science, as well as the motivation for and actual performance of several non-recommended prevention behaviors (Dohle et al., 2020; Imhoff and Lamberty, 2020). These questions were assessed for exploratory purposes and are not discussed further.

### 2.3. Data analysis

Data was analyzed separately for each of the DHM-behaviors. To test the hypothesized direct and indirect causal effects of coping and threat appraisals on behavior, all variables were analyzed as latent variables using a two-step structural equation modeling (SEM) approach (Anderson and Gerbing, 1988). We first performed confirmatory factor analyses (CFA) to ensure adequate measurement of the latent constructs. Following a partial adjustment of the measurement models, we then examined the proposed structural relationships of the variables.

Reliability and validity of the measurement models were assessed using established criteria including the squared standardized factor loadings (squared multiple correlations, *SMC*s ≥ 0.4 indicate good indicator reliability; Weiber and Mühlhaus, 2014), Raykov’s rho coefficient (ρ >0.7 indicates good construct reliability; Raykov, 2004; Hair et al., 2013), standardized factor loadings (values >0.5 indicate adequate, values >0.7 good convergent validity; Hair et al., 2013), and estimated factor correlations (absolute values <0.85 indicate adequate discriminant validity in factor measurement; Brown, 2006). Moreover, a variety of fit indices was employed to evaluate model fit. In addition to the inferential Chi-square test (Bollen-Stine *p* value >0.05 indicates good exact fit) we report the chi-square to degrees of freedom ratio (absolute values 2 and 3 indicate an adequate fit; Schreiber et al., 2006), the Root Mean Square Error of Approximation (RMSEA, values ≤0.05 and those ranging from 0.05─0.08, respectively, indicate an good and adequate fit; Browne and Cudeck, 1992), the Standardized Root Mean Square Residual (SRMR; common cut-off ≤0.08 for model acceptance; Hu and Bentler, 1999) and the Comparative Fit Index (CFI; ≥ 0.90 recommended for the given sample size and high model complexity; Hair et al., 2013). In addition to global fit measures, we examined standardized residuals to identify local areas of misfit at the level of pairs of observed variables (values | ≥ 2.58| indicate a large deviation of model-implied covariances from those obtained empirically; Kline, 2005; Byrne, 2013).

Consistent with our pre-registration,^3^ path coefficients significant at *p* < 0.05 were considered to support direct and indirect effects of the appraisal variables as hypothesized by Rogers (1975, 1983). In an exploratory part of our analyses, we estimated alternative models including two additional direct paths (costs ➔ behavior; self-efficacy ➔ behavior). Added paths were trimmed if their estimated coefficients were not significant. The resulting alternative structures to the original PMT models were retained if Chi-square-difference tests and information criteria (AIC; Akaike’s information criterion; and Bayesian information criterion; BIC) suggested their superiority: A significant chi-square difference statistic indicates that the hypothesis of equal model fit needs to be rejected as the addition of free parameters (i.e., the direct paths) significantly improved the correspondence of the model with the data (Kline, 2005). In using AIC and BIC for model comparison, we include model parsimony as an additional criterion to the goodness of fit to the data (Kline, 2005).

### 2.4. Data screening

Data preparation and calculation of descriptive statistics were performed using R version 4.0.0 and SPSS version 27. Participants remaining at T2 were significantly older (*M* = 45.63, *SD* = 14.28) than those individuals who responded only at T1 (*M* = 37.18, *SD* = 13.95), *t*(317 = 3.86, *p* < 0.001, *d* = 0.59). Furthermore, they were slightly less likely to report having a significant other belonging to a vulnerable risk group, X^2^ (1) = 5.54, *p =* 0.019, Φ = 0.14. Despite these two effects, no differences were found between the initial and final samples in terms of demographics or any of our study variables. Furthermore, Little’s *MCAR* Test (Little, 1988) was nonsignificant, X^2^ (60) = 76.79, *p =* 0.071, suggesting that observed missing data patterns can be assumed to be missing completely at random (Hair et al., 2013). Prior to SEM analyses and for each behavior, the dataset was evaluated for multivariate outliers by examining squared Mahalanobis distances (*D^2^*). Deleting cases with a distinctively large *D^2^* (Byrne, 2013; Weiber and Mühlhaus, 2014) left us with sample sizes of *N* = 275 (physical distancing and mask wearing) and *N* = 271 (hand hygiene). SEM was performed with AMOS version 27 using maximum-likelihood estimation. Skewness and kurtosis of a number of indicators and Mardia testing (Mardia, 1970) indicated non-normality in the data. This was addressed in all our subsequent analyses by performing bootstrapping (5,000 samples) to derive bootstrap standard errors as well as bias-corrected confidence intervals for all parameter estimates and Bollen-Stine *p*-values for model testing.

## 3. Results

### 3.1. Evaluation of the measurement model

Based on protection motivation theory, we specified three CFA-models (physical distancing, hand hygiene, wearing a mask) with eight factors each. The number of indicators loading on the six appraisal variables, protection motivation and protective behavior varied from 3─5 (see Table 1). All latent variables were permitted to be correlated, all measurement errors were presumed to be uncorrelated, and all indicators were specified to load onto a single factor only. Latent variables were scaled by imposing unit loading identification constraints, meaning that all unstandardized residual path coefficients were fixed to equal 1.0, while the same was done with the factor loading of one indicator per factor (Kline, 2005). We further established identification by fulfilling the requirements of overidentification (*df* ≥ 0 in all our models) and having more than two indicators per factor (Kline, 2005).

Even though our initial measurement models demonstrated adequate fit with respect to most metrics (see Supplementary Table A5), modification indices (> 4.0) provided by AMOS prompted us to make several adjustments to improve model fit. All modifications of our initial measurement models are documented in the Supplementary material for this article (Supplementary Tables A2–A4) alongside with our rationale underlying these respecifications. Here, we provide a summary of all indicators used in the final measurement models including a documentation of those that we deleted. Graphical representations (Supplementary Figure A1) depict which error covariances have been added. The different fit indices (also reported in Supplementary Table A5) indicated model improvement, with most of them attesting adequate to good fit following our adjustments. Detailed CFA results including interfactor correlations, standardized and unstandardized parameter estimates can be found in the Supplementary Tables A6–A9. Reliability and validity analyses yielded satisfactory results (factor loadings of the individual items 0.59─0.98, all *p* ≤ 0.001; *SMC* 0.35─0.95; construct reliabilities ρ = 0.73─0.93). As an exception, we found the factors self-efficacy and intention to be highly correlated (*r* = 0.86─0.90, *p* < 0.001) in two of the models. As this observation is in line with the assumption of self-efficacy being the most important predictor of protection motivation (Milne et al., 2000), we kept measurements of these variables distinct.

### 3.2. Hypothesis testing

**Figure 1:**
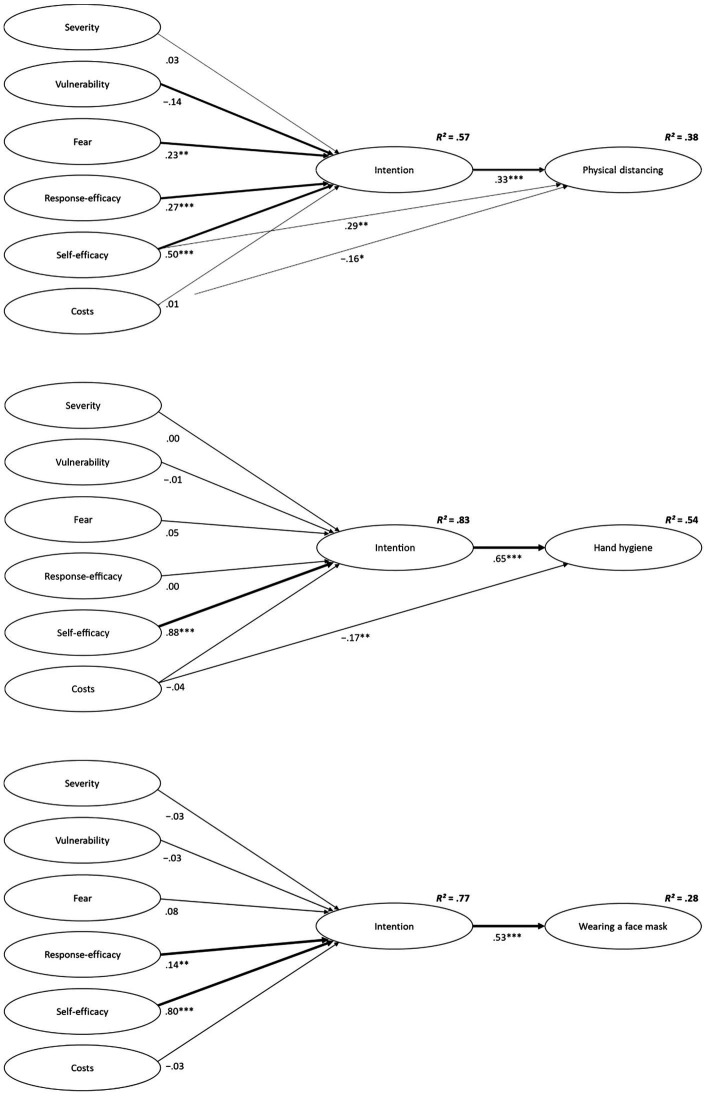
Results from the finally retained structural equation models (physical distancing and mask wearing: *N* = 275; hand hygiene: *N* = 271) depicting associations between appraisal variables, intentions, and protective behavior. Standardized beta coefficients are shown. Bold lines indicate significant indirect effects. *p < 0.05; **p < 0.01; ***p < 0.001.

In the second part of our analyses, the three final measurement models derived from confirmatory factor analyses were respecified as a fully latent SEMs incorporating direct and indirect effects as proposed by PMT. As fit indices listed in Table 2 indicate, these models demonstrated acceptable to good fit to the data. As a second step, each of them was compared to our proposed alternative models. Results from the modified and finally retained models are depicted in Figure 1.

**Table 2:** Comparison of fit indices in models fitted according to PMT and our proposed alternative theoretical model.

Model	*χ* ^2^						RMSEA			CFI	SRMR	AIC	BIC
	*χ* ^2^	*df*	Bollen-Stine-p	∆χ^2^	∆*df*	∆ *p*	RMSEA	90% CI	*p*				
Distancing (*N* = 275)													
1. Initial model	728.22	376	< 0.001				0.058	[0.052, 0.065]	0.015	0.962	0.069	906.215	1228.108
**2. Added pathways:** **S-E → D** **Costs → D**	**701.81**	**374**	**0.001**	26.41***	**2**	**<0.001**	**0.057**	**[0.050, 0.063]**	**0.048**	**0.964**	**0.062**	**883.810**	**1212.937**
Hygiene (*N* = 271)													
1. Initial model	969.347	376	< 0.001				0.076	[0.071, 0.082]	0.000	0.942	0.063	1147.347	1467.936
**2. Added pathway:** **Costs → H**	**960.004**	**375**	**< 0.001**	9.343**	**1**	**0.002**	**0.076**	**[0.070, 0.082]**	**0.000**	**0.943**	**0.060**	**1140.004**	**1464.195**
3. Added pathways: S-E ➔ H Costs ➔ H	959.997	374	< 0.001	0.007	1	0.933	0.076	[0.070, 0.082]	0.000	0.943	0.060	1141.997	1469.790
Mask wearing (*N* = 275)													
**1. Initial model**	**528.749**	**273**	**0.002**	8.307**	1	0.004	**0.058**	**[0.051, 0.066]**	**0.032**	**0.973**	**0.043**	**684.749**	**966.857**
2. Added pathways: S-E ➔ M Costs ➔ M	520.293	271	0.002	0.149	1	0.699	0.058	[0.050, 0.065]	0.042	0.973	0.041	680.293	969.634

Model alternatives printed in bold were retained. RMSEA, Root Mean Square Error of Approximation. CFI, Comparative Fit Index; SRMR, Standardized Root Mean Square Residual; AIC, Akaike Information Criterion; BIC, Bayesian Information Criterion; S-E, Self-efficacy; D, Physical distancing; H, Hand hygiene; M, Wearing of a hygienically clean face mask. **p < 0.01, **p < 0.001.

#### 3.2.1. Physical distancing

In the original PMT model (Model 1), coping and threat variables accounted for 57% of the variance of distancing intentions, whereas intentions accounted for 30% of the performed behavior. In line with theoretical assumptions, higher levels of fear (*β* = 0.23, 95% CI [0.09; 0.38], *p* = 0.004), perceived response efficacy (*β* = 0.27, 95% CI [0.14; 0.39], *p* < 0.001), and self-efficacy (*β* = 0.50, 95% CI [0.38; 0.63], *p* < 0.001) predicted heightened intentions to adhere to the measure. In addition, individuals with higher intentions (*β* = 0.55, 95% CI [0.42; 0.66], *p* < 0.001) showed physical distancing more frequently in the following month. Furthermore, fear (*b* = 0.07, 95% CI [0.03; 0.13], *p* = 0.003), response efficacy (*b* = 0.11, 95% CI [0.06; 0.17], *p* < 0.001), and self-efficacy (*b* = 0.17, 95% CI [0.11; 0.24], *p* < 0.001), had significant indirect effects on behavior *via* intention. Replicating previous PMT research [4], the effects of coping appraisal variables were higher in magnitude than were those of threat appraisal variables, with self-efficacy being the strongest predictor overall. Hypotheses on perceived severity and costs were not supported; both proved to be neither predictive of distancing intentions (severity: *β* = 0.03, 95% CI [−0.11; 16], *p* = 0.699; costs: *β* = 0.00, 95% CI [−0.08; 0.09], *p* = 0.886) nor indirectly related to performed distancing behavior (severity: *b* = 0.01, 95% CI [−0.04; 0.07], *p =* 0.690; costs: *b* = 0.00, 95% CI [−0.04; 0.04], *p =* 0.884). Considering oneself as highly vulnerable for an infection with COVID-19 was negatively related to distancing intentions, (*β* = −0.14, 95% CI [−0.26, −0.03], *p* = 0.012) and indirectly predicted less adherence to this measure, (*b* = −0.06, 95% CI [−0.12; −0.02], *p* = 0.009).

##### 3.2.1.1. Alternative theoretical model

Looking at the prediction of physical distancing in the original (Model 1) vs. alternative model (Model 2), a significant Chi-square-difference test and reduced AIC and BIC values (see Table 2) indicated improvement through the inclusion of the additional pathways. Including the two direct paths increased the proportion of explained variance in behavior by 8%. Results show that self-efficacy is not only indirectly related to more frequent physical distancing (i.e., *via* heightened intentions), *b* = 0.10, 95% CI [0.05; 0.17], *p* < 0.001, but also directly predictive of this behavior, *β* = 0.29, 95% CI [0.11; 0.45], *p* = 0.002. For costs, we again found no indication that they were directly (*β* = 0.01, 95% CI [−0.08; 0.10], *p* = 0.784) linked to intentions or indirectly (*b* = 0.00, 95% CI [−0.02; 0.03], *p* = 0.745) linked to behavior *via* intentions. However, we found evidence for a direct effect on physical distancing, *β* = −0.16, 95% CI [−0.29; −0.04], *p* = 0.011. That is, independent of their intentions stated at T1 measurement, participants who rated the measure as being more costly (T1) reported less adherence to physical distancing recommendations later (T2).

#### 3.2.2. Hand hygiene

The analysis revealed that the original PMT model (Model 1) explained 83% of the variance in intentions to perform hand hygiene and 52% of the variance in the behavior. The high level of explained variance, however, was primarily due to self-efficacy: Self-efficacy was the only variable significantly associated with heightened intentions to adhere to the measure (*β* = 0.88, 95% CI [0.78; 0.97], *p* < 0.001) and, indirectly *via* its positive effect on intentions, higher frequency of performed behavior, (*b* = 0.48, 95% CI [0.34; 0.62], *p* < 0.001).

##### 3.2.2.1. Alternative theoretical model

Concerning the prediction of performed hand hygiene, the included link between costs and behavior was significant, *β* = −0.17, 95% CI [−0.27; −0.06], *p =* 0.004, while the one between self-efficacy and behavior was not, *p =* 0.891 (Model 3). As removing the latter did not cause a loss in model fit, a second alternative (Model 2) was retained representing the original PMT structure extended by the additional costs-behavior pathway only. Compared to the original model (Model 1), including this path slightly increased explained variance in hand hygiene from 52 to 54%. Again, perceived costs were directly related to less protective behavior, *β* = −0.17, 95% CI [−0.27; −0.06], *p =* 0.004, while there was no evidence of an effect on intentions, *p =* 0.378, or an indirect effect on behavior mediated by intentions, *p =* 0.349.

#### 3.2.3. Mask wearing

The proportion of variance in outcomes explained by the original PMT model (Model 1) was 77% for intentions to wear a face mask in the next month and 28% for the reported frequency of measure adoption. Theoretical assumptions were supported for both response efficacy and self-efficacy. That is, the belief about the effectiveness of mask wearing in reducing the risk of an infection as well as the belief in being capable to do so in different circumstances had a direct association with heightened behavioral intentions (response efficacy: *β* = 0.14, 95% CI [0.04; 0.26], *p* = 0.008; self-efficacy: *β* = 0.80, 95% CI [0.70; 0.88], *p* < 0.001) and an indirect association with greater frequencies of measure adoption (response efficacy: *b* = 0.05, 95% CI [0.02; 0.11], *p* = 0.005; self-efficacy: *b* = 0.28, 95% CI [0.18; 0.42], *p* < 0.001). Again, self-efficacy was shown to be the stronger predictor among the two.

##### 3.2.3.1. Alternative theoretical model

The additional direct effects were not significant in the alternative model estimated to predict mask wearing, even though results seemed somewhat more supportive of the proposed self-efficacy-behavior link (Model 2; costs ➔ behavior, *p* = 0.718, self-efficacy ➔ behavior, *p* = 0.161). Hence, the original PMT model (Model 1) was retained for the prediction of hygiene behavior.

## 4. Discussion

Disease containment during the current and future pandemics will heavily rely on citizens’ motivation to adopt protective measures and their actual behavior in everyday life. The main aim of this research was to identify determinants of the DHM-behaviors (keeping physical distance, hand hygiene, wearing a facemask) recommended in the context of COVID-19. Using a prospective design in a representative national sample, we proved Rogers’ protection motivation theory to be a viable framework to explain intentions and subsequent protective behavior of the German adult population. Our analyses point to the higher relative relevance of coping appraisal, as compared to threat appraisal: Testing the original theoretical model, we found higher self-efficacy to be indirectly predictive of better adherence to all DHM-measures *via* increased intentions. Albeit smaller in magnitude, response efficacy also had a positive indirect effect on two out of three examined behaviors. Contrary to the findings of other studies, which were conducted in the first wave of the COVID-19 pandemic (Kaspar, 2020; Coifman et al., 2021; Kowalski and Black, 2021), our data (collected in the second wave in Germany) failed to confirm the hypothesized effects of perceived severity. Higher levels of fear arousal were predictive of higher intentions and (indirectly) better adherence to one protective measure (physical distancing) only. These results suggest that, once a population habituates to a new health threat, risk perceptions cease to be a major determinant of performed protective behavior. Future longitudinal research should examine such dynamics over prolonged periods in the course of evolving pandemics. For now, we conclude that health campaigns aimed at promoting public adherence should benefit from fostering individuals’ self-efficacy (i.e., strengthening their belief in their capability to adhere even in challenging situations) and perceived response efficacy of the DHM-behaviors (i.e., emphasizing their effectiveness in reducing the transmission risk). Importantly, when compared to risk perceptions, perceived trust in the government and public health authorities have been shown to be equally relevant or an even stronger predictor of adherence during pandemics (Bults et al., 2011; Dohle et al., 2020). Thus, even if informing the public about the disease severity and arising emotions of fear may motivate individuals to take preventive action, sustainable changes should require building lasting trust through accuracy and an open addressing of scientific uncertainties (Bults et al., 2011).

As common in health behavior research (Sheeran, 2002), the results indicated a substantive lack of consistency in intentions reported at baseline (57–83% of variance explained by the three models) and subsequently reported behavior (28–52% of variance explained). One implication is that interventions targeting change in the proposed intention determinants (e.g., perceived response efficacy) will affect behavioral enactment to a markedly lesser extent (Hagger et al., 2020). In that respect, our exploratory analyses yielded promising results: The inclusion of additional direct paths into our alternative theoretical model improved the prediction of adherence and model fit in all three cases. Both perceived self-efficacy and costs were shown to be directly predictive of some of the DHM-behaviors. Instead of merely having an influence through the process of intention (as hypothesized by PMT), these perceptions thus may also determine health preventive action immediately. Interestingly, we found heterogeneities between the different models predicting each of the three behaviors: Both hypothesized direct effects were found most consistently for physical distancing, while results on hand hygiene and mask wearing appear less clear cut. A similar finding was reported in a PMT-based study on adherence to a gluten-free diet among patients with celiac disease (Dowd et al., 2016), where self-efficacy was directly predictive of accidental gluten ingestion, but not of intentional consumption. Keeping a distance to other people, just as avoiding accidential incidents of gluten consumptions, might represent comparatively challenging target behaviors that are beyond complete volitional control of the acting persons (Ajzen, 1991; Fishbein and Ajzen, 2009). It seems plausible that the two direct effects reflect that implementing positive intentions requires continuous effort and persistence, making it crucial for individuals to believe in the feasibility of reaching their goal and their ability to attain it (Dowd et al., 2016). Besides this psychological effect, low self-efficacy and high perceived costs might indicate that there are non-psychological barriers present actually limiting the feasibility of following prevention guidelines. Supporting this interpretation, many participants left a comment on our survey stating that it had been impossible for them to maintain a distance to other people in crowded places. Moreover, recent studies found low socioeconomic status and belonging to an ethnic minority to relate to worse adherence and greater intention-behavior gaps concerning COVID-19 infection protection (Atchison et al., 2021; Gibson et al., 2021). Recent survey data from the UK suggests that those with low incomes and savings were equally willing to self-isolate as their more affluent counterparts, yet less likely to do so (Atchison et al., 2021). Pointing to a lack of opportunities as a root cause of socioeconomic disparities in actual behavior, the financially disadvantaged reported more frequently that they were unable to work from home, i.e., due to a lack of permission or necessary equipment, or to self-isolate if needed (Atchison et al., 2021).

The finding of a direct effect from self-efficacy on behavior corrobates basic assumptions of other social cognition theories and matches the results of other PMT-based studies (Fishbein and Ajzen, 2009; Conner and Norman, 2015; Dowd et al., 2016). Considering perceived costs of the different measures, our findings expand previous theoretical and empirical work related to PMT. Contrary to the assumptions of PMT, costs did not predict intentions in any of the models, but had a significant, negative effect on performed physical distancing and hand hygiene behaviors. Therefore, costs do not seem to discourage people from aiming to adopt protective measures, yet to hinder them from actually doing so. As outlined above, insurmountable obstacles can prevent even highly motivated individuals from taking action. Another explanation might be that the DHM-measures appear rather easy to implement when being considered in the survey situation, whereas discomfort, inconvenience or disruption of daily activities can powerfully change momentary preferences when being experienced (Loewenstein, 2005; Nordgren et al., 2008). The observed pattern might therefore reflect that risky behaviors in the pandemic occur from a failure of self-control when individuals experience immediate impulses that are contrary to their long-term health goal of avoiding an infection (Loewenstein, 1996, 2005). The strong average intentions to adhere to the DHM-measures and the intention-behavior gap displayed by our sample point to another important implication. At this stage of the pandemic, it would be beneficial for public health interventions to increase emphasis on supporting (already motivated) individuals in translating their intentions into actual behavior change. Given their supposed direct effects on infection protection behavior, it seems particularly viable to target individuals’ self-efficacy, perceptions of costs and to foster their self-regulatory abilities to overcome even strong habitual responses or impulses (Gibson et al., 2021). In addition, intervention efforts should aim at reducing actual barriers and providing facilitating environments where possible (Teasdale et al., 2012).

This conclusion is consistent with the implication of studies on hand hygiene among healthcare workers conducted in the two decades prior to the COVID-19 pandemic: In the face of various perceived costs, i.e., time lost due to hand washing under the burden of high work load in the hospital environment (Pittet et al., 2000; Reichardt et al., 2009), self-efficacy and perceived ease of hand hygiene interventions were found to be particularly important determinants of compliance (Sax et al., 2007; De Wandel et al., 2010; Erasmus et al., 2020). Again, researchers have emphasized that supporting hospital staff to implement their positive intentions is crucial (Reichardt et al., 2009). Interventions have achieved positive changes in compliance, particularly by increasing the immediate availability of alcoholic hand rubs as a time-saving alternative to soap and water hand washing (Pittet et al., 2000). Our findings suggest that in the general population, adherence to hand hygiene follows similar mechanisms and that multimodal intervention strategies should target the individual and environmental levels (Pittet et al., 2000).

The findings presented in this report are subject to certain limitations. First, cross-validation or replication using data from a new sample is warranted to confirm our exploratory model modifications and forward theoretical development (Kline, 2005). Secondly and albeit anonymization, our participant’s self-reports might have been subject to social desirability effects and problems of recall. Next, even though the temporal sequence of our measurements of appraisal variables and behavior is a key strength of this study, the observed correlations do not necessarily imply a causal relationship. Moreover, as appraisal variables and intentions were assessed cross-sectionally, the direction of potential effects remains unclear (Brewer et al., 2004). Two of the SEM analyses revealed nonsignificant effects of vulnerability on intentions, while the physical distancing model pointed to a small negative effect. This heterogeneity might result from some participants assessing their personal risk based on an anticipation of their planned preventive action (producing a negative correlation among the two) while others did so independently from their intentions (Farin, 1994; Brewer et al., 2004). Even though we do not find clear evidence of a motivational effect of vulnerability, we thus refrain from drawing definite conclusions on its absence. Furthermore, legal measures implemented during the time of our study (e.g., mask requirements and regulations on social gatherings in public, Schuppert et al., 2021) made adherence compulsory, suggesting that all appraisal variables may have had stronger effects on protective behaviors under less regulated conditions.

While the threat appraisal variables did not have an effect on either intentions or behaviors in the models predicting hand hygiene or mask wearing, they were significant predictors in the physical distancing model. It is important to note that in the former two models, the correlation of self-efficacy and intentions to adhere was strongly positive. Proponents of the *self-efficacy-as-motivation argument* argue that items commonly used to measure self-efficacy often actually measure participants’ motivation instead of their perceived capability, especially when the behavior in question is easy to control (Williams and Rhodes, 2016). If this was the case for our measurements regarding hand washing and mask wearing, including self-efficacy as a predictor in the models might have masked the effects of the other appraisal variables (Williams and Rhodes, 2016). Importantly, this concern points to the need to re-evaluate the role of self-efficacy in the general study of health behaviors using alternative operationalizations (Williams and Rhodes, 2016).

Another reason for the weak evidence of a motivational effect of perceived risk on protective behaviors might be that risky behavior during a pandemic is not only consequential for the acting individual, but also for others and society as a whole (Hagger et al., 2020). Focusing on PMT as our explanatory model, we have omitted other variables─i.e., subjective norms, moral obligations, and empathic responding to other people feeling threatened─which may also elicit preventive behaviors even if an individual does not feel personally threatened (Hagger et al., 2020; Morstead et al., 2021). Besides considering other direct predictors of heath protective behaviors, future work should investigate variables that might moderate or mediate the intention-behavior relationship (e.g., use of self-regulation strategies or availability of resources) and can be targeted to support individuals to follow through with their intentions (Hagger et al., 2020; Gibson et al., 2021).

### 4.1. Conclusion

As novel emerging infectious diseases will pose us with new challenges in the future, it will most likely remain central to identify determinants of the public’s adherence to prevention guidelines and thereby find targets for behavioral interventions. The result of this research suggests that motivation and actual behavior depend on individuals’ self-efficacy, as well as perceived and actually existent barriers to adherence. We conclude that during a pandemic, policymakers and public health institutions should not only provide information about the risks and consequences of viral infection. Instead, they should support individuals in developing self-efficacy and address perceptions of difficulties and costs, while also providing them with resources, opportunities and environmental conditions that actually facilitate the adoption of protective measures (Teasdale et al., 2012; Hagger et al., 2020).

## Data availability statement

The datasets presented in this study can be found in online repositories. The names of the repository/repositories and accession number(s) can be found below: https://osf.io/yx8aw/?view_only=34ab39a433e646fb95ebff675b9543ce (OSF). This study was pre-registered on AsPredicted (available at https://aspredicted.org/547kp.pdf).

## Ethics statement

The studies involving human participants were reviewed and approved by Faculty of Human Sciences of the University of Cologne. The patient/participant provided their written informed consent to participate in this study.

## Author contributions

MH contributed to conceptualization, data curation, formal analysis, methodology, project administration, and writing–original draft, review, and editing. SD contributed to conceptualization, data curation, methodology, funding acquisition, project administration, and writing–review and editing. All authors contributed to the article and approved the submitted version.

## Funding

This work was supported by a grant from the University of Cologne Center of Excellence for Social and Economic Behavior (C-SEB) awarded to SD. This work was supported by the Open Access Publication Fund of the University of Bonn.

## Conflict of interest

The authors declare that the research was conducted in the absence of any commercial or financial relationships that could be construed as a potential conflict of interest.

## Publisher’s note

All claims expressed in this article are solely those of the authors and do not necessarily represent those of their affiliated organizations, or those of the publisher, the editors and the reviewers. Any product that may be evaluated in this article, or claim that may be made by its manufacturer, is not guaranteed or endorsed by the publisher.

## Footnotes

^1^ https://www.respondi.com

^2^ https://www.qualtrics.com

^3^ https://aspredicted.org/GFO_RCI
