# Supplementary material for: Personal protective behaviors in response to COVID-19: a longitudinal application of protection motivation theory
Source: Front Psychol. 2023 Aug 15;14:1195607. doi: 10.3389/fpsyg.2023.1195607 (PMC10465328; doi:10.3389/fpsyg.2023.1195607)
Supplement: Supplementary file 1 [file Table_1.DOC]

**Supplemental material**

**Table A1**

*Sociodemographic Characteristics of the Study Population at T1 (N = 328) and T2 (N* = 278).

|  | T1 | | | | T2 | | | |
| --- | --- | --- | --- | --- | --- | --- | --- | --- |
| Variable | *M* | *SD* | *n* | % | *M* | *SD* | *n* | *%* |
| Age | 44.39 | 14.57 |  |  | 45.83 | 14.32 |  |  |
| Age group |  |  |  |  |  |  |  |  |
| 18-29 |  |  | 67 | 20.4 |  |  | 48 | 17.3 |
| 30-39 |  |  | 62 | 18.9 |  |  | 48 | 17.3 |
| 40-49 |  |  | 61 | 18.6 |  |  | 55 | 19.8 |
| 50-59 |  |  | 77 | 23.5 |  |  | 67 | 24.1 |
| 60-69 |  |  | 61 | 18.6 |  |  | 60 | 21.6 |
| Gender |  |  |  |  |  |  |  |  |
| Male |  |  | 164 | 50 |  |  | 137 | 49.3 |
| Female |  |  | 164 | 50 |  |  | 141 | 50.7 |
| Subjective health | 3.68 | 0.84 |  |  | 3.65 | 0.84 |  |  |
| Subjective health status |  |  |  |  |  |  |  |  |
| 1 very bad |  |  | 6 | 1.8 |  |  | 5 | 1.8 |
| 2 bad |  |  | 17 | 5.2 |  |  | 16 | 5.8 |
| 3 fair |  |  | 96 | 29.3 |  |  | 85 | 30.6 |
| 4 good |  |  | 165 | 50.3 |  |  | 136 | 48.9 |
| 5 very good |  |  | 44 | 13.4 |  |  | 36 | 13 |
| Vulnerable risk group |  |  |  |  |  |  |  |  |
| 1 yes |  |  | 106 | 32.3 |  |  | 94 | 33.81 |
| 2 no |  |  | 222 | 67.7 |  |  | 184 | 66.19 |
| Significant other in risk group |  |  |  |  |  |  |  |  |
| 1 yes |  |  | 170 | 51.8 |  |  | 136 | 48.9 |
| 2 no |  |  | 158 | 48.2 |  |  | 142 | 51.1 |

**Table A2**

*Physical distancing: content for incl*uded and deleted survey items

| Construct |
| --- |
| Severity  SV_1 Severity of general consequences if contracting the coronavirus  SV_2 Seriousness of general consequences if contracting the coronavirus  SV_3 Severity of health consequences if contracting the coronavirus  SV_4 Severity of social consequences if contracting the coronavirus |
| Vulnerability  VL_1 Great likelihood of contracting the coronavirus until the end of the year  VL_2 High risk of contracting the coronavirus until the end of the year  VL_3 High likelihood of contracting the coronavirus until the end of the year  VL_4 Highly at risk for contracting the coronavirus until the end of the year *(deleted)* |
| Fear  FR_1 Worried about the possibility of contracting the coronavirus  FR_2 Bothered by the thought of contracting the coronavirus  FR_3 Worried by the idea of contracting the coronavirus  FR_4 Scared by the thought of contracting the coronavirus |
| Response-efficacy  RE_D1 In public transport *(deleted)*  RE_D2 In supermarkets / other stores  RE_D3 Outdoors in public (on the street / pedestrian zone / at public places)  RE_D4 In public buildings  RE_D5 At longer meetings with other people from other households |
| Self-efficacy  SE_D1 In public transport *(deleted)*  SE_D2 In supermarkets / other stores  SE_D3 Outdoors in public (on the street / pedestrian zone / at public places) SE_D4 In public buildings  SE_D5 At longer meetings with other people from other households |
| Costs  CO_D1 Can cause difficulties  CO_D2 Is uncomfortable  CO_D3 Is exhausting |
| Intention  PM_D1 In public transport *(deleted)*  PM_D2 In supermarkets / other stores  PM_D3 Outdoors in public (on the street / pedestrian zone / at public places) PM_D4 In public buildings  PM_D5 At longer meetings with other people from other households |
| Behavior  PB_D1 In public transport *(deleted)*  PB_D2 In supermarkets / other stores  PB_D3 Outdoors in public (on the street / pedestrian zone / at public places) PB_D4 In public buildings  PB_D5 At longer meetings with other people from other households |

*Note.* *N* = 275.

Item VL_4 was removed due to potential cross-loadings indicated by modification indices.

Item PB_D1 was removed due to low indicator reliability (*SMC* = .391). As 19 participants commented on our survey stating that they either never used public transport or find it impossible to keep distance in crowded busses or trains, it seems likely that ratings on this item do not provide a valid measure of protective behavior. As a consequence of the item’s removal, the corresponding items on response-efficacy, self-efficacy and intentions concerning physical distancing in public transport were deleted as well.

**Table A3**

*Hand hygiene: content for included and deleted survey items*

| Construct |
| --- |
| Severity  SV_1 Severity of general consequences if contracting the coronavirus  SV_2 Seriousness of general consequences if contracting the coronavirus  SV_3 Severity of health consequences if contracting the coronavirus  SV_4 Severity of social consequences if contracting the coronavirus |
| Vulnerability  VL_1 Great likelihood of contracting the coronavirus until the end of the year  VL_2 High risk of contracting the coronavirus until the end of the year  VL_3 High likelihood of contracting the coronavirus until the end of the year  VL_4 Highly at risk for contracting the coronavirus until the end of the year *(deleted)* |
| Fear  FR_1 Worried about the possibility of contracting the coronavirus  FR_2 Bothered by the thought of contracting the coronavirus  FR_3 Worried by the idea of contracting the coronavirus  FR_4 Scared by the thought of contracting the coronavirus |
| Response-efficacy  RE_H1 After coming home  RE_H2 Before and after eating and drinking in private space  RE_H3 Before food preparation  RE_H4 Before and after making contact with others |
| Self-efficacy  SE_H1 After coming home  SE_H2 Before and after eating and drinking in private space  SE_H3 Before food preparation  SE_H4 Before and after making contact with others |
| Costs  CO_H1 Can cause difficulties  CO_H2 Is uncomfortable  CO_H3 Is exhausting |
| Intention  PM_H1 After coming home  PM_H2 Before and after eating and drinking in private space  PM_H3 Before food preparation  PM_H4 Before and after making contact with others |
| Behavior  PB_H1 After coming home  PB_H2 Before and after eating and drinking in private space  PB_H3 Before food preparation  PB_H4 Before and after making contact with others |

*Note.* *N* = 271.

Item VL_4 was removed due to potential cross-loadings indicated by modification indices.

**Table A4**

*Wearing a hygienically clean face mask: content for included and deleted survey items*

| Construct |
| --- |
| Severity  SV_1 Severity of general consequences if contracting the coronavirus  SV_2 Seriousness of general consequences if contracting the coronavirus  SV_3 Severity of health consequences if contracting the coronavirus  SV_4 Severity of social consequences if contracting the coronavirus |
| Vulnerability  VL_1 Great likelihood of contracting the coronavirus until the end of the year  VL_2 High risk of contracting the coronavirus until the end of the year  VL_3 High likelihood of contracting the coronavirus until the end of the year  VL_4 Highly at risk for contracting the coronavirus until the end of the year *(deleted)* |
| Fear  FR_1 Worried about the possibility of contracting the coronavirus  FR_2 Bothered by the thought of contracting the coronavirus  FR_3 Worried by the idea of contracting the coronavirus  FR_4 Scared by the thought of contracting the coronavirus |
| Response-efficacy  RE_M1 In public transport  RE_M2 In supermarkets / other stores  RE_M3 In public buildings  RE_M4 At longer meetings with other people from other households *(deleted)* |
| Self-efficacy  SE_M1 In public transport  SE_M2 In supermarkets / other stores  SE_M3 In public buildings  SE_M4 At longer meetings with other people from other households *(deleted)* |
| Costs  CO_M1 Can cause difficulties  CO_M2 Is uncomfortable  CO_M3 Is exhausting |
| Intention  PM_M1 In public transport  PM_M2 In supermarkets / other stores  PM_M3 In public buildings  PM_M4 At longer meetings with other people from other households *(deleted)* |
| Behavior  PB_M1 In public transport  PB_M2 In supermarkets / other stores  PB_M3 In public buildings  PB_M4 At longer meetings with other people from other households *(deleted)* |

*Note.* *N* = 275.

Item VL_4 was removed due to potential cross-loadings indicated by modification indices.

Item PB_M4 was removed due to low indicator reliability (*SMC* = .292), probably resulting from its diverging content (referring to specific situation / activity rather than a specific location). As a consequence of the item’s removal, the corresponding items on response-efficacy, self-efficacy and intentions concerning mask wearing at meetings with other people were deleted as well.

**Table A5**

*Results from Confirmatory Factor* Analyses: Initial and modified measurement models

| Measurement model | χ² | | |  |  | RMSEA |  | CFI | SRMR | AIC | BIC |
| --- | --- | --- | --- | --- | --- | --- | --- | --- | --- | --- | --- |
|  | χ² | *df* | Bollen-Stine- *p* |  | RMSEA | 90% KI | *p* |  |  |  |  |
| Distancing |  |  |  |  |  |  |  |  |  |  |  |
| 1. Initial | 1197.87 | 532 | <.001 |  | .068 | [.062, .073] | <.001 | .938 | .062 | 1393.874 | 1748.318 |
| 2. Modified | 697.224 | 370 | .001 |  | .057 | [.050; .063] | .043 | .964 | .059 | 887.224 | 1230.817 |
|  |  |  |  |  |  |  |  |  |  |  |  |
| Hand hygiene |  |  |  |  |  |  |  |  |  |  |  |
| 1. Initial | 1475.418 | 406 | <.001 |  | .099 | [.093, .104] | <.001 | .899 | .065 | 1655.418 | 1979.609 |
| 2. Modified | 955.485 | 370 | <.001 |  | .077 | [.071; .083] | <.001 | .943 | .061 | 1145.485 | 1487.686 |
|  |  |  |  |  |  |  |  |  |  |  |  |
| Mask wearing |  |  |  |  |  |  |  |  |  |  |  |
| 1. Initial | 1211.28 | 406 | <.001 |  | .085 | [.080, .091] | <.001 | .926 | .064 | 1391.28 | 1716.789 |
| 2. Modified | 513.332 | 267 | .002 |  | .058 | [.050; .066] | .041 | .974 | .038 | 681.332 | 985.141 |

*Note:* Analyses on physical distancing and mask wearing based on *N* = 275, those on hand hygiene behavior based on *N* = 271. RMSEA = Root Mean Square Error of Approximation; CFI = Comparative Fit Index; SRMR = Standardized Root Mean Square Residual; AIC = Akaike Information Criterion; BIC = Bayesian Information Criterion.

**Table A6**

*Confirmatory Factor Analysis Results for Physical distancing*: Estimated Factor Loadings, Indicator Reliabilities and Error Variances

|  |  | Unstd.  *(SE)* | *p* | Std. | *SMC* | Error variance *(SE)* | *p* |
| --- | --- | --- | --- | --- | --- | --- | --- |
|  | Severity |  |  |  |  |  |  |
|  | SV_1 | 1.00 (–) |  | 0.91 | .83 | 0.44 (0.07) | <.001 |
|  | SV_2 | 1.00 (0.03) | <.001 | 0.89 | .80 | 0.57 (0.11) | <.001 |
|  | SV_3 | 1.10 (0.03) | <.001 | 0.97 | .95 | 0.16 (0.05) | <.001 |
|  | SV_4 | 1.05 (0.03) | <.001 | 0.95 | .91 | 0.26 (0.05) | <.001 |
|  | Vulnerability |  |  |  |  |  |  |
|  | VL_1 | 1.00 (–) |  | 0.90 | .82 | 0.38 (0.07) | <.001 |
|  | VL_2 | 1.06 (0.06) | <.001 | 0.88 | .78 | 0.53 (0.15) | <.001 |
|  | VL_3 | 1.13 (0.05) | <.001 | 0.97 | .94 | 0.14 (0.03) | <.001 |
|  | Fear |  |  |  |  |  |  |
|  | FR_1 | 1.00 (–) |  | 0.98 | .95 | 0.18 (0.04) | <.001 |
|  | FR_2 | 0.98 (0.02) | <.001 | 0.95 | .91 | 0.33 (0.06) | <.001 |
|  | FR_3 | 0.99 (0.02) | .001 | 0.97 | .94 | 0.22 (0.04) | <.001 |
|  | FR_4 | 1.00 (0.01) | <.001 | 0.98 | .95 | 0.18 (0.04) | <.001 |
|  | Response-efficacy |  |  |  |  |  |  |
|  | RE_D2 | 1.00 (–) |  | 0.94 | .89 | 0.27 (0.09) | <.001 |
|  | RE_D3 | 0.85 (0.04) | <.001 | 0.81 | .65 | 0.86 (0.17) | <.001 |
|  | RE_D4 | 1.05 (0.03) | <.001 | 0.98 | .95 | 0.12 (0.06) | .027 |
|  | RE_D5 | 1.02 (0.05) | <.001 | 0.88 | .77 | 0.67 (0.11) | <.001 |
|  | Self-efficacy |  |  |  |  |  |  |
|  | SE_D2 | 1.00 (–) |  | 0.91 | .82 | 0.66 (0.10) | <.001 |
|  | SE_D3 | 0.93 (0.05) | <.001 | 0.88 | .77 | 0.80 (0.18) | <.001 |
|  | SE_D4 | 0.94 (0.04) | .001 | 0.91 | .83 | 0.57 (0.14) | <.001 |
|  | SE_D5 | 0.86 (0.05) | <.001 | 0.81 | .65 | 1.24 (0.20) | <.001 |
|  | Costs |  |  |  |  |  |  |
|  | CO_D1 | 1.00 (–) |  | 0.65 | .42 | 2.17 (0.27) | <.001 |
|  | CO_D2 | 0.91 (0.12) | <.001 | 0.59 | .35 | 2.46 (0.22) | <.001 |
|  | CO_D3 | 1.58 (0.22) | <.001 | 0.95 | .91 | 0.41 (0.43) | .327 |
|  | Intention |  |  |  |  |  |  |
|  | PM_D2 | 1.00 (–) |  | 0.94 | .88 | 0.28 (0.07) | <.001 |
|  | PM_D3 | 1.00 (0.04) | .001 | 0.87 | .76 | 0.61 (0.16) | <.001 |
|  | PM_D4 | 0.98 (0.03) | <.001 | 0.94 | .89 | 0.23 (0.05) | <.001 |
|  | PM_D5 | 0.90 (0.06) | <.001 | 0.71 | .50 | 1.56 (0.24) | <.001 |
|  | Behavior |  |  |  |  |  |  |
|  | PB_D2 | 1.00 (–) |  | 0.86 | .74 | 0.41 (0.09) | <.001 |
|  | PB_D3 | 1.00 (0.06) | <.001 | 0.83 | .68 | 0.54 (0.14) | <.001 |
|  | PB_D4 | 1.01 (0.05) | <.001 | 0.85 | .72 | 0.46 (0.14) | <.001 |
|  | PB_D5 | 0.90 (0.08) | <.001 | 0.66 | .44 | 1.20 (0.19) | <.001 |

*Note.* *N* = 275. *SMC* = Squared Multiple Correlation. Unstd. = Unstandardized factor loadings.
Std. = Standardized factor loadings.

**Table A7**

*Confirmatory Factor Anal*ysis Results for Hand hygiene: Estimated Factor Loadings, Indicator Reliabilities and Error Variances

|  | Unstd.  *(SE)* | *p* | Std. | *SMC* | Error variance  *(SE)* | *p* |
| --- | --- | --- | --- | --- | --- | --- |
| Severity |  |  |  |  |  |  |
| SV_1 | 1.00 (–) |  | 0.91 | 0.83 | 0.45 (0.07) | <.001 |
| SV_2 | 0.98 (0.03) | <.001 | 0.88 | 0.78 | 0.60 (0.11) | <.001 |
| SV_3 | 1.10 (0.03) | <.001 | 0.97 | 0.95 | 0.15 (0.05) | <.001 |
| SV_4 | 1.06 (0.03) | <.001 | 0.95 | 0.91 | 0.24 (0.04) | <.001 |
| Vulnerability |  |  |  |  |  |  |
| VL_1 | 1.00 (–) |  | 0.90 | 0.81 | 0.39 (0.07) | <.001 |
| VL_2 | 1.08 (0.05) | <.001 | 0.91 | 0.83 | 0.40 (0.10) | <.001 |
| VL_3 | 1.13 (0.05) | <.001 | 0.97 | 0.94 | 0.14 (0.03) | <.001 |
| Fear |  |  |  |  |  |  |
| FR_1 | 1.00 (–) |  | 0.98 | 0.95 | 0.17 (0.04) | <.001 |
| FR_2 | 0.98 (0.02) | <.001 | 0.95 | 0.90 | 0.34 (0.06) | <.001 |
| FR_3 | 0.99 (0.02) | <.001 | 0.97 | 0.94 | 0.19 (0.03) | <.001 |
| FR_4 | 1.00 (0.01) | <.001 | 0.98 | 0.95 | 0.17 (0.04) | <.001 |
| Response-efficacy |  |  |  |  |  |  |
| RE_H1 | 1.00 (–) |  | 0.93 | 0.86 | 0.30 (0.16) | <.001 |
| RE_H2 | 1.04 (0.11) | <.001 | 0.86 | 0.74 | 0.68 (0.24) | <.001 |
| RE_H3 | 1.03 (0.10) | <.001 | 0.89 | 0.79 | 0.51 (0.18) | <.001 |
| RE_H4 | 0.98 (0.03) | <.001 | 0.90 | 0.81 | 0.41 (0.17) | <.001 |
| Self-efficacy |  |  |  |  |  |  |
| SE_H1 | 1.00 (–) |  | 0.86 | 0.74 | 0.57 (0.10) | <.001 |
| SE_H2 | 1.24 (0.09) | <.001 | 0.97 | 0.94 | 0.16 (0.04) | <.001 |
| SE_H3 | 1.20 (0.08) | <.001 | 0.97 | 0.93 | 0.17 (0.04) | <.001 |
| SE_H4 | 0.94 (0.06) | <.001 | 0.71 | 0.50 | 1.42 (0.22) | <.001 |
| Costs |  |  |  |  |  |  |
| Cost_H1 | 1.00 (–) |  | 0.75 | 0.57 | 1.66 (0.27) | <.001 |
| Cost_H2 | 1.01 (0.08) | <.001 | 0.83 | 0.69 | 0.99 (0.17) | <.001 |
| Cost_H3 | 1.26 (0.10) | <.001 | 0.92 | 0.84 | 0.65 (0.26) | .011 |
| Intention |  |  |  |  |  |  |
| PM_H1 | 1.00 (–) |  | 0.82 | 0.67 | 0.62 (0.11) | <.001 |
| PM_H2 | 1.43 (0.13) | <.001 | 0.95 | 0.90 | 0.27 (0.07) | <.001 |
| PM_H3 | 1.32 (0.12) | <.001 | 0.94 | 0.88 | 0.30 (0.08) | <.001 |
| PM_H4 | 1.08 (0.06) | <.001 | 0.79 | 0.62 | 0.90 (0.15) | <.001 |
| Behavior |  |  |  |  |  |  |
| PB_H1 | 1.00 (–) |  | 0.72 | 0.53 | 0.83 (0.14) | <.001 |
| PB_H2 | 1.42 (0.20) | <.001 | 0.91 | 0.82 | 0.41 (0.09) | <.001 |
| PB_H3 | 1.39 (0.20) | <.001 | 0.90 | 0.81 | 0.42 (0.08) | <.001 |
| PB_H4 | 1.09 (0.09) | <.001 | 0.71 | 0.51 | 1.05 (0.20) | <.001 |

*Note.* *N* = 271. *SMC* = Squared Multiple Correlation. Unstd. = Unstandardized factor loadings.
Std. = Standardized factor loadings.

**Table A8**

*Confirmatory Factor Analysis Results for Mask Wearing: Estimated Factor Loadings, Indicator R*eliabilities and Error Variances

|  | Unstd.  *(SE)* | *p* | Std. | *SMC* | Error variance  *(SE)* | *p* |
| --- | --- | --- | --- | --- | --- | --- |
| Severity |  |  |  |  |  |  |
| SV_1 | 1.00 (–) |  | 0.91 | 0.83 | 0.46 (0.07) | <.001 |
| SV_2 | 0.99 (0.03) | .001 | 0.88 | 0.78 | 0.61 (0.11) | <.001 |
| SV_3 | 1.10 (0.03) | <.001 | 0.97 | 0.95 | 0.15 (0.04) | <.001 |
| SV_4 | 1.06 (0.03) | <.001 | 0.96 | 0.91 | 0.24 (0.04) | <.001 |
| Vulnerability |  |  |  |  |  |  |
| VL_1 | 1.00 (–) |  | 0.90 | 0.82 | 0.38 (0.07) | <.001 |
| VL_2 | 1.06 (0.06) | <.001 | 0.88 | 0.78 | 0.54 (0.15) | <.001 |
| VL_3 | 1.12 (0.05) | <.001 | 0.96 | 0.92 | 0.18 (0.05) | <.001 |
| Fear |  |  |  |  |  |  |
| FR_1 | 1.00 (–) |  | 0.97 | 0.95 | 0.19 (0.04) | <.001 |
| FR_2 | 0.98 (0.02) | <.001 | 0.95 | 0.90 | 0.35 (0.07) | <.001 |
| FR_3 | 0.98 (0.02) | .001 | 0.97 | 0.93 | 0.23 (0.04) | <.001 |
| FR_4 | 1.00 (0.01) | <.001 | 0.98 | 0.95 | 0.17 (0.04) | <.001 |
| Response-efficacy |  |  |  |  |  |  |
| RE_M1 | 1.00 (–) |  | 0.92 | 0.85 | 0.44 (0.10) | <.001 |
| RE_M2 | 1.01 (0.03) | <.001 | 0.97 | 0.94 | 0.16 (0.05) | <.001 |
| RE_M3 | 1.02 (0.03) | <.001 | 0.98 | 0.96 | 0.10 (0.05) | .009 |
| Self-efficacy |  |  |  |  |  |  |
| SE_M1 | 1.00 (–) |  | 0.93 | 0.86 | 0.43 (0.17) | <.001 |
| SE_M2 | 0.97 (0.03) | .001 | 0.98 | 0.96 | 0.10 (0.04) | .002 |
| SE_M3 | 0.96 (0.03) | .001 | 0.96 | 0.93 | 0.19 (0.06) | <.001 |
| Costs |  |  |  |  |  |  |
| Cost_M1 | 1.00 (–) |  | 0.84 | 0.70 | 1.43 (0.28) | <.001 |
| Cost_M2 | 1.07 (0.06) | <.001 | 0.92 | 0.84 | 0.75 (0.18) | <.001 |
| Cost_M3 | 1.01 (0.06) | <.001 | 0.85 | 0.73 | 1.30 (0.22) | <.001 |
| Intention |  |  |  |  |  |  |
| PM_M1 | 1.00 (–) |  | 0.90 | 0.81 | 0.46 (0.18) | <.001 |
| PM_M2 | 0.97 (0.04) | .001 | 0.97 | 0.95 | 0.10 (0.04) | .002 |
| PM_M3 | 0.93 (0.04) | .001 | 0.95 | 0.91 | 0.17 (0.06) | <.001 |
| Behavior |  |  |  |  |  |  |
| PB_M1 | 1.00 (–) |  | 0.68 | 0.46 | 1.34 (0.38) | <.001 |
| PB_M2 | 0.64 (0.15) | .001 | 0.78 | 0.60 | 0.30 (0.10) | <.001 |
| PB_M3 | 0.98 (0.10) | .001 | 0.86 | 0.74 | 0.40 (0.13) | .001 |

*Note.* *N* = 275. *SMC* = Squared Multiple Correlation. Unstd. = Unstandardized factor loadings.
Std. = Standardized factor loadings.

**Table A9**

*Results of Confirmatory Factor Analyses: Interf*actor correlations

| Variable | 1 | 2 | 3 | 4 | 5 | 6 | 7 | 8 |
| --- | --- | --- | --- | --- | --- | --- | --- | --- |
|  | Physical distancing | | | | | | | |
| 1. Severity | 1.00 |  |  |  |  |  |  |  |
| 2. Vulnerability | .44*** | 1.00 |  |  |  |  |  |  |
| 3. Fear | .78*** | .49*** | 1.00 |  |  |  |  |  |
| 4. Response-efficacy | .20** | .04 | .19** | 1.00 |  |  |  |  |
| 5. Self-efficacy | .16* | –.05 | .16* | .50*** | 1.00 |  |  |  |
| 6. Costs | .07 | .08 | .09 | –.02 | –.25*** | 1.00 |  |  |
| 7. Intention | .27*** | –.03 | .31*** | .56*** | .68** | –.11 | 1.00 |  |
| 8. Behavior | .20** | –.04 | .20** | .28*** | .55** | –.27*** | .54*** | 1.00 |
|  | Hand hygiene | | | | | | | |
| 1. Severity | 1.00 |  |  |  |  |  |  |  |
| 2. Vulnerability | .44*** | 1.00 |  |  |  |  |  |  |
| 3. Fear | .78*** | .49*** | 1.00 |  |  |  |  |  |
| 4. Response-efficacy | .29*** | .05 | .25*** | 1.00 |  |  |  |  |
| 5. Self-efficacy | .25*** | .06 | .19** | .65*** | 1.00 |  |  |  |
| 6. Costs | –.07 | .01 | –.01 | –.23*** | –.42*** | 1.00 |  |  |
| 7. Intention | .26*** | .07 | .22** | .60*** | .91*** | –.41*** | 1.00 |  |
| 8. Behavior | .12 | –.01 | .09 | .36*** | .66*** | –.43*** | .72*** | 1.00 |
|  | Wearing a hygienically clean face mask | | | | | | | |
| 1. Severity | 1.00 |  |  |  |  |  |  |  |
| 2. Vulnerability | .43*** | 1.00 |  |  |  |  |  |  |
| 3. Fear | .78*** | .48*** | 1.00 |  |  |  |  |  |
| 4. Response-efficacy | .32*** | .15 | .37*** | 1.00 |  |  |  |  |
| 5. Self-efficacy | .25*** | .10 | .27*** | .46*** | 1.00 |  |  |  |
| 6. Costs | –.13 | –.11 | –.17* | –.42*** | –.26*** | 1.00 |  |  |
| 7. Intention | .26*** | .09 | .31*** | .51*** | .86*** | –.24*** | 1.00 |  |
| 8. Behavior | .19* | .13* | .21* | .40*** | .54*** | –.17* | .52*** | 1.00 |

*Note.* Interfactor correlations for physical distancing and mask wearing based on *N* = 275, those for hand hygiene behavior based on *N* = 271.[[1]](#footnote-2)
**p* < .05. ***p* < .01. ****p* < .001.

**Figure A1**


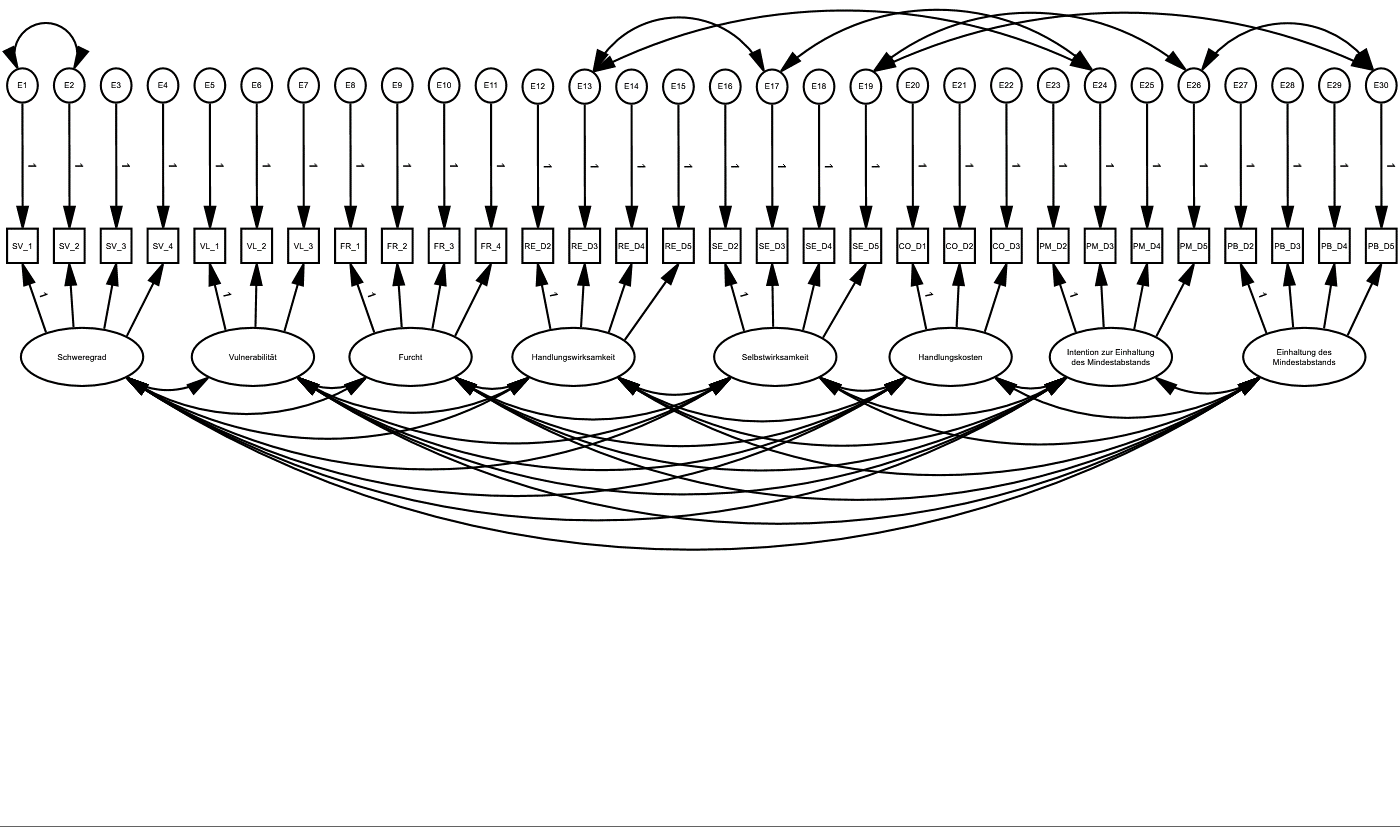
**A**

*
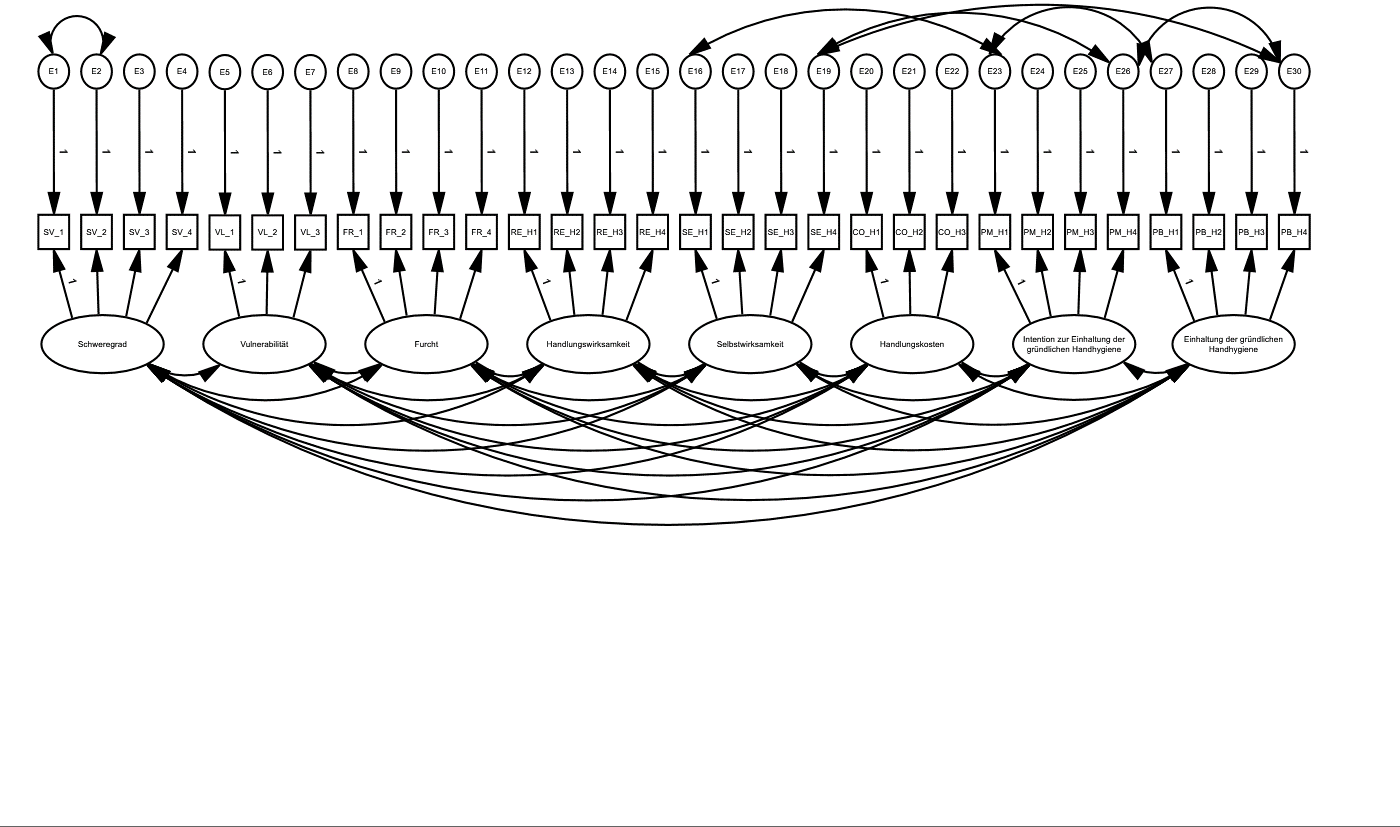
*

**B**


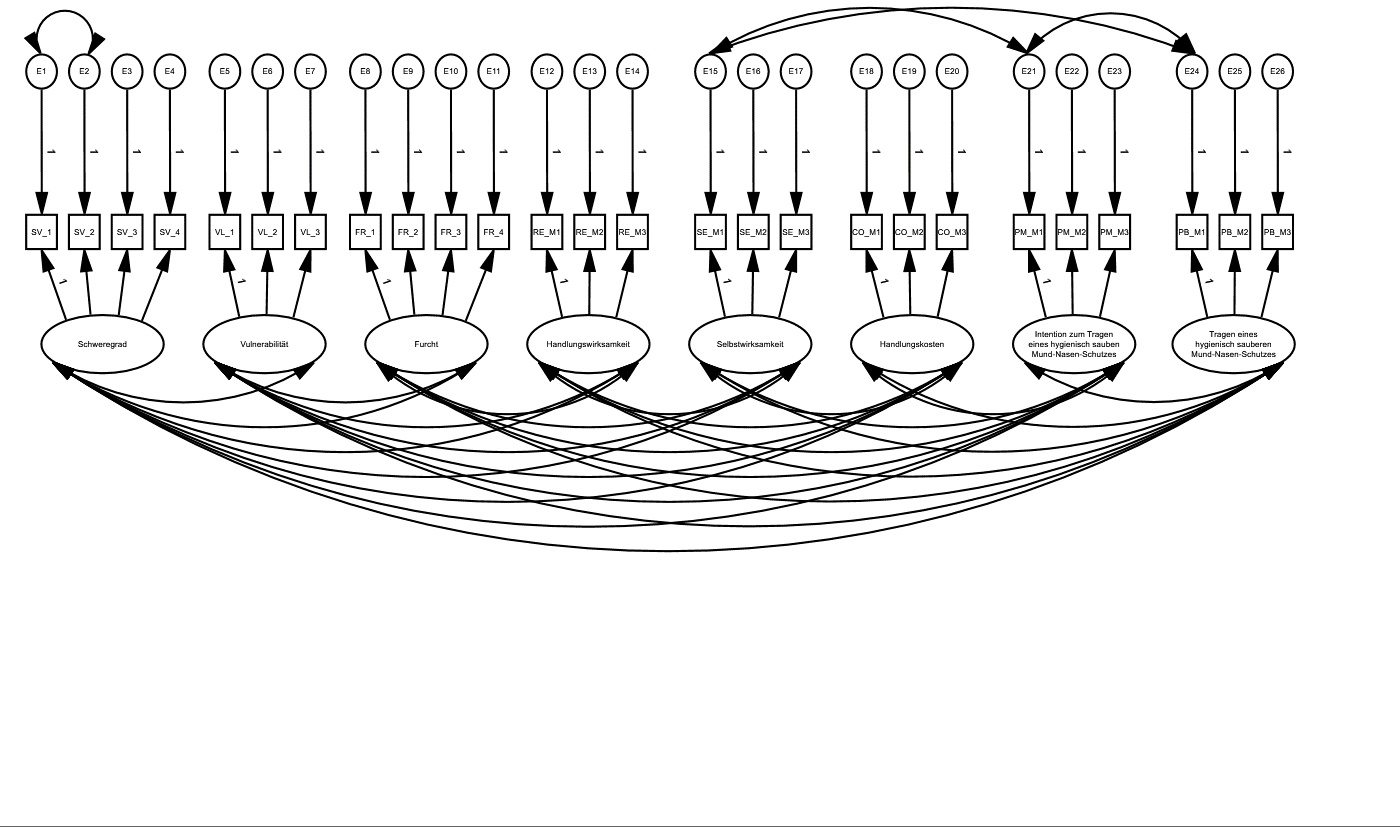
C

Modified Measurement Models with Added Error Covariances. All estimated error covariances were significant at *p* < .05. In all measurement models (A-C), error covariances were added between the following items: SV1_2 and SV_2. Those items share the equal wording, both referring to „general consequences“ of an infection.

In measurement model A (physical distancing), further error covariances were added between the following items: RE_D3 and SE_D3, RE_D3 and PM_D3, SE_D3 and PM_D3 (all refering to physical distancing in the same situation [outdoors in public]); SE_D5 and PM_D5, SE_D5 and PB_D5, PM_D5 and PB_D5 (all refering to the same situation [at longer meetings]).

In measurement model B (hand hygiene), further error covariances were added between the following items: SE_H1 and PM_H1, SE_H1 and PB_H1, PM_H1 and PB_H1 (all referring to the same situation [after coming home]). SE_H4 and PM_H4, SE_H4 and PB_H4, PM_H4 and PB_H4 (all referring to the same situation [before and after making contact with others]).

In measurement model C (mask wearing), further error covariances were added between the following items: SE_M1 and PM_M1, SE_M1 and PB_M1, PM_M1 and PB_M1 (all referring to the same situation [in public transport]).

**References**

Cohen, J. (1988). *Statistical power analysis for the behavioral sciences* (2nd ed.). Routledge.

Floyd, D. L., Prentice-Dunn, S., & Rogers, R. W. (2000). A meta-analysis of research on protection motivation theory. *Journal of Applied Social Psychology*, *30*(2), 407–429. https://doi.org/10.1111/j.1559-1816.2000.tb02323.x

Milne, S., Sheeran, P., & Orbell, S. (2000). Prediction and intervention in health-related behavior: A meta-analytic review of protection motivation theory. *Journal of Applied Social Psychology*, *30*(1), 106–143. https://doi.org/10.1111/j.1559-1816.2000.tb02308.x

Rogers, R. W. (1975). A protection motivation theory of fear appeals and attitude change. *The Journal of Psychology*, *91*(1), 93–114. https://doi.org/10.1080/00223980.1975.9915803

Rogers, R. W. (1983). Cognitive and physiological processes in fear appeals and attitude change: A revised theory of protection motivation. In R. E. Petty & J. T. Cacioppo (Eds.), *Social psychophysiology: A sourcebook* (pp. 153–176). Guilford Press.

1. Intercorrelations generally support the relationships as hypothesized by Rogers (1975, 1983): On a bivariate level, severity, fear, response efficacy and self-efficacy are positively related to behavioral intentions and behaviors, while perceived costs were negatively related to the named variables. In addition, behavioral intentions were strongly related to actual performed protective behaviors (Cohen, 1988). Notably, factor correlations between perceived vulnerability and intentions / behavior were nonsignificant and near zero in all but one case (significant positive correlation with reported frequency of mask wearing), thus failing to support the supposed positive associations.

   In line with previous work (Floyd et al., 2000; Milne et al., 2000), effects were found to be stronger for coping appraisals as compared to threat appraisals. Also, our results replicate the finding of correlations between appraisals and intentions being generally stronger than those between appraisals and behavior (Milne et al., 2000). The associations between perceived costs and behavior, however, were found to be either comparable to those between costs and intention (hand hygiene and mask wearing models) or even stronger than the latter (physical distancing model). [↑](#footnote-ref-2)
